# Potential Distribution Prediction of *Terminalia chebula* Retz. in China Under Current and Future Climate Scenarios

**DOI:** 10.1002/ece3.70908

**Published:** 2025-01-28

**Authors:** Zhang‐Hong Dong, Hua Jiang, Wei Zhang, Jianhua Wu, Yanping Yang, Taoming Yang, Jiangping Zhao, Cunzhen Luo, Xiaoxia Yang, Guilin Li

**Affiliations:** ^1^ Forestry and Grassland Technique Extention Station of Baoshan City Baoshan China; ^2^ Forestry and Grassland Scientific Research Institute of Baoshan City Baoshan China

**Keywords:** climate change, habitat suitability, MaxEnt model, species distribution, *Terminalia chebula*

## Abstract

Climate change in the future could potentially expand, shrink, or alter the habitats of numerous species, leading to changes in their spatial distributions. Predicting suitable areas for cultivating medicinal plants through modeling has become an effective tool for assessing site suitability and conserving medicinal plant resources. Utilizing GIS and MaxEnt model, we predicted the spatial distribution of *Terminalia chebula* Retz. in China for the current and for the future (2050s and 2070s) under the RCP4.5 and RCP8.5 representative concentration pathways. In this study, we utilized 73 occurrence records and incorporated eight environmental factors from WorldClim for the modeling process. The findings revealed that the evaluation of the model's performance was based on the area under the curve (AUC) of the receiver operating characteristic (ROC). All AUC values exceeded 0.9, classifying these models as “Excellent.” Additionally, the jackknife test analysis revealed that the main influential variables were bio11 and bio4. Under the present climate conditions, the estimated total suitable habitat for *T. chebula* is approximately 29.14 × 10^4^ km^2^, representing around 2.78% of China's total land area. Within these suitable regions, high suitability, medium suitability, and low suitability areas make up 0.39%, 0.54%, and 1.85% of the total area, respectively. According to future climate, the potential growth range of *T. chebula* is expected to expand due to climate variability, showing a significant pattern of expansion towards the north and east within China. In the 2050s and 2070s, the total area of regions with high suitability, medium suitability, and low suitability under RCP4.5 and RCP8.5 will increase compared to the current distribution. This study will provide theoretical suggestions for preservation, management, and sustainable utilization of *T. chebula* resources.

## Graphical abstract

According to future climate, the potential growth range of *T. chebula* is expected to expand due to climate variability, showing a significant pattern of expansion towards the north and east within China. In the 2050s and 2070s, the total area of regions with high suitability, medium suitability, and low suitability under RCP4.5 will increase compared to the current distribution.

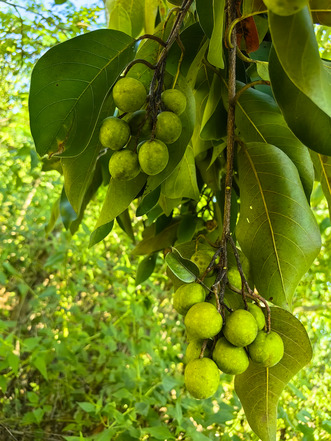

## 1 Introduction

Climate change is driving the spread of plants and causing changes in the range of suitable growing areas (Hebbar et al. 2022; Li et al. 2019). In the decades ahead, biodiversity will face a greater threat from global warming than from habitat destruction (Bradshaw et al. 2021). Climate significantly influences plant growth and reproduction, playing a major role in determining the geographical distribution of plant species (Bertrand et al. 2011; Lenoir et al. 2008). Due to the increasing impact of climate change and human activities, as well as the more frequent occurrence of unusual weather patterns at a regional level, there have been notable shifts in the geographic distribution of many species (Ceballos et al. 2015; Sanderson et al. 2002). This has had a certain influence on plants distribution (Aguirre‐Liguori et al. 2019; Coumou and Rahmstorf 2012; Ramírez‐Preciado, Gasca‐Pineda, and Arteaga 2019). Climate change is causing medicinal plant habitats to shrink or move, endangering their sustainable use (Barnosky et al. 2011; Rana et al. 2020; Shen, Yu, and Wang 2021). Therefore, it is crucial to investigate how plants are affected by climate change to protect them (Gupta et al. 2021; Wang et al. 2023a; Zhang et al. 2019). Using ecological models to forecast suitable habitat locations, determine optimal planting areas, and analyze key environmental factors impacting habitat distribution is essential.

Climate plays a crucial role in population regeneration, growth, and the distribution of species (Pyšek et al. 2020; Zhang, Li, and Du 2018). Previous research has demonstrated that both temperature and precipitation significantly influence plant growth, survival, and reproduction (Poncet et al. 2010). These climatic factors indirectly influence the spatial distribution of species by modulating their phenological and physiological processes (Manzoor, Griffiths, and Lukac 2021; Marchioro and Krechemer 2021). Furthermore, global climate change and extreme weather events have the potential to drastically alter the spatial distribution and prevalence of species. Consequently, investigating the spatial patterns of suitable habitats for medicinal plants in the context of future climate change is essential for effective risk assessment and management. With the advancement of statistical models and geographic information systems (GIS), ecological theory and GIS technology have been extensively used in various fields, including ecology, protection, and development (Brito et al. 2009; Guisan and Zimmermann 2000; Warren et al. 2008). Currently, species distribution modeling (SDM) is the most commonly used method for studying potential species distribution and environmentally suitable habitats (Booth 2016; Booth et al. 2014; Elith and Leathwick 2009). The popular species distribution models include bioclimatic modeling (BIOCLIM), climate change experiment (CLIMEX), domain environmental envelope (Domain), ecological niche factor analysis (ENFA), genetic algorithm for rule‐set production (GARP), and maximum entropy (MaxEnt). MaxEnt is considered the most effective method in species distribution modeling (SDM) approaches because of its superior effectiveness and outstanding outcomes (Elith et al. 2006), even with a limited sample size (Pearson and Dawson 2003). The MaxEnt model uses species distribution location data and environmental information to approximate the probability distribution with the highest entropy value. This allows for the analysis and prediction of possible geographical distribution patterns of various species (Phillips and Dudík 2008). The model not only combines numerous environmental variables in its distribution predictions but also pinpoints key influential factors and the extent of adaptation impacting species growth (Wang et al. 2023a). Commonly employed for both forecasting species distribution and researching ecological properties, the MaxEnt model is indispensable in scientific research (Shen, Yu, and Wang 2021; Wang et al. 2023a; Zeng et al. 2021).

*Terminalia chebula* Retz. is a member of the Combretaceae family and a traditional Chinese medicinal plant belonging to the *Terminalia* L. genus. It is primarily found in South and Southeast Asian countries, where it is mostly humid tropical and subtropical. Its main distribution is in Baoshan, Dehong, and Lincang in the southwest of China. Wild populations of *T. chebula* were found in the Nujiang Basin and its tributaries (Kui and Luo 2010). As an effective astringent, dried fruit of *T. chebula* has a long history of traditional medicine for the treatment of many diseases, including cardiovascular disorders, tumors, gastrointestinal disorders, skin diseases, and diabetes (Barthakur and Arnold 1991; Na Takuathung et al. 2023; Singh et al. 1978). *T. chebula* has been reported to contain a diverse chemical composition, including phenolic acids, tannins, triterpenoids, aliphatics, flavonoids, volatile oils, amino acids, trace elements, carbohydrates, and so on (Zhao et al. 2020). Modern pharmacological research indicates that *T. chebula* extract exhibits a variety of pharmacological activities, including antioxidation (Hazra et al. 2010; Saha and Verma 2016), anticancer (Kumar et al. 2014), antitumor (Athira et al. 2013; Lu et al. 2012), detoxification (Wang, Zhang, and Zhai 2002; Yang et al. 2013), antibacterial (Rekha et al. 2014; Sarabhai, Sharma, and Capalash 2013; Zhong et al. 2014), immunomodulation (Das et al. 2012; Shin et al. 2001), and promoting bronchial smooth muscle contraction (Pang et al. 2001). As a traditional Chinese medicine, *T. chebula* has not carried out related research on habitat suitability.

*Terminalia chebula* is a valuable medicinal plant; however, the potential impact of climate change on its distribution remains uncertain. Therefore, it is essential to study how plants respond to climate change to ensure their conservation, growth, and utilization. This study used the MaxEnt method to predict the impact of climate change on the distribution of *T. chebula* . The findings of the research illustrate the current and future geographical distribution patterns of the plant under different climatic conditions, providing a scientific foundation for the sustainable use and management of *T. chebula* resources.

## 2 Materials and Methods

### 2.1 Collection of Species Geographical Distribution Data

**FIGURE 1:**
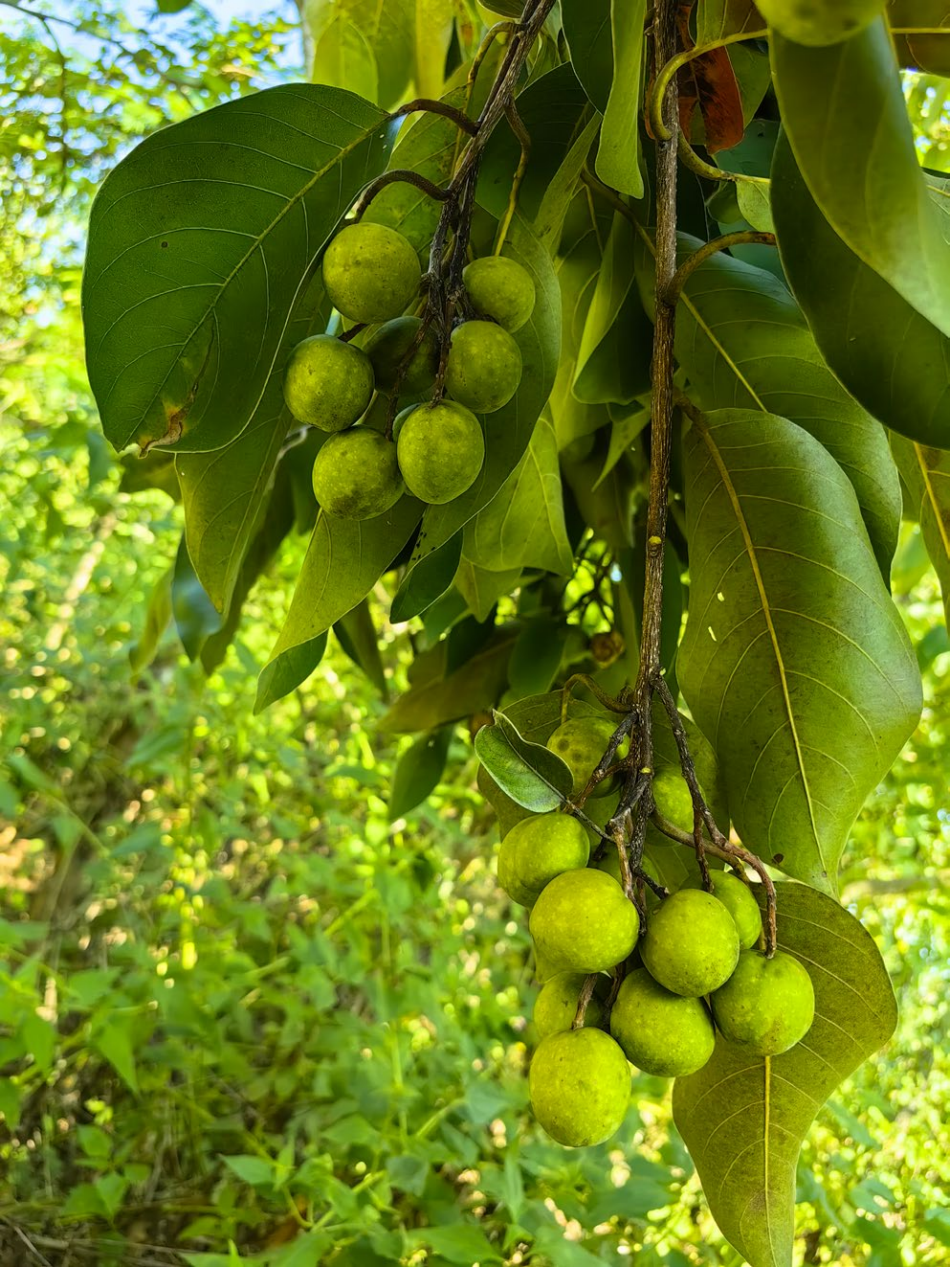
Biological characteristics of *T. chebula* plant.

**FIGURE 2:**
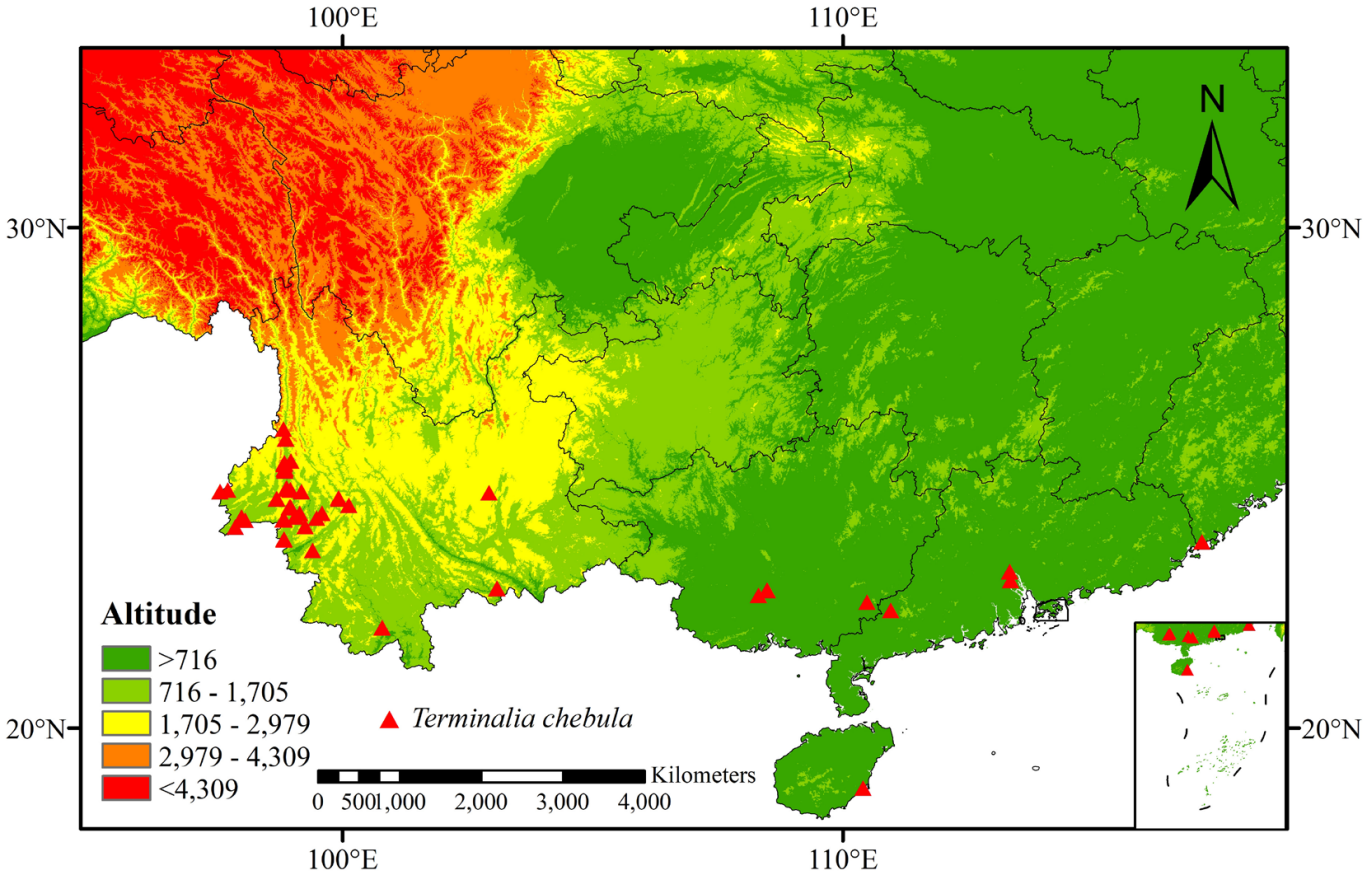
Spatial distribution of occurrence records of *T. chebula* in China.

The spatial distribution data of *T. chebula* were obtained from the Global Biodiversity Information Facility (GBIF; https://www.gbif.org/), Chinese Virtual Herbarium (CVH; https://www.cvh.ac.cn/), National Specimen Information Infrastructure (NSII; http://www.nsii.org.cn/), literature sources, and data from 236 field sampling sites collected during germplasm resource collection with the research group. Plant characteristics are as shown in Figure 1. Based on the above data, we accurately determined latitude and longitude. We retained 292 samples after removing duplicate and inaccurate records (Table S1). In order to minimize sampling bias, we filtered the distribution points of the initial data using ENMtools (Warren et al. 2008) software. Following the filtering, we ended up with 73 valid samples (Figure 2).

### 2.2 Environmental Variables

**FIGURE 3:**
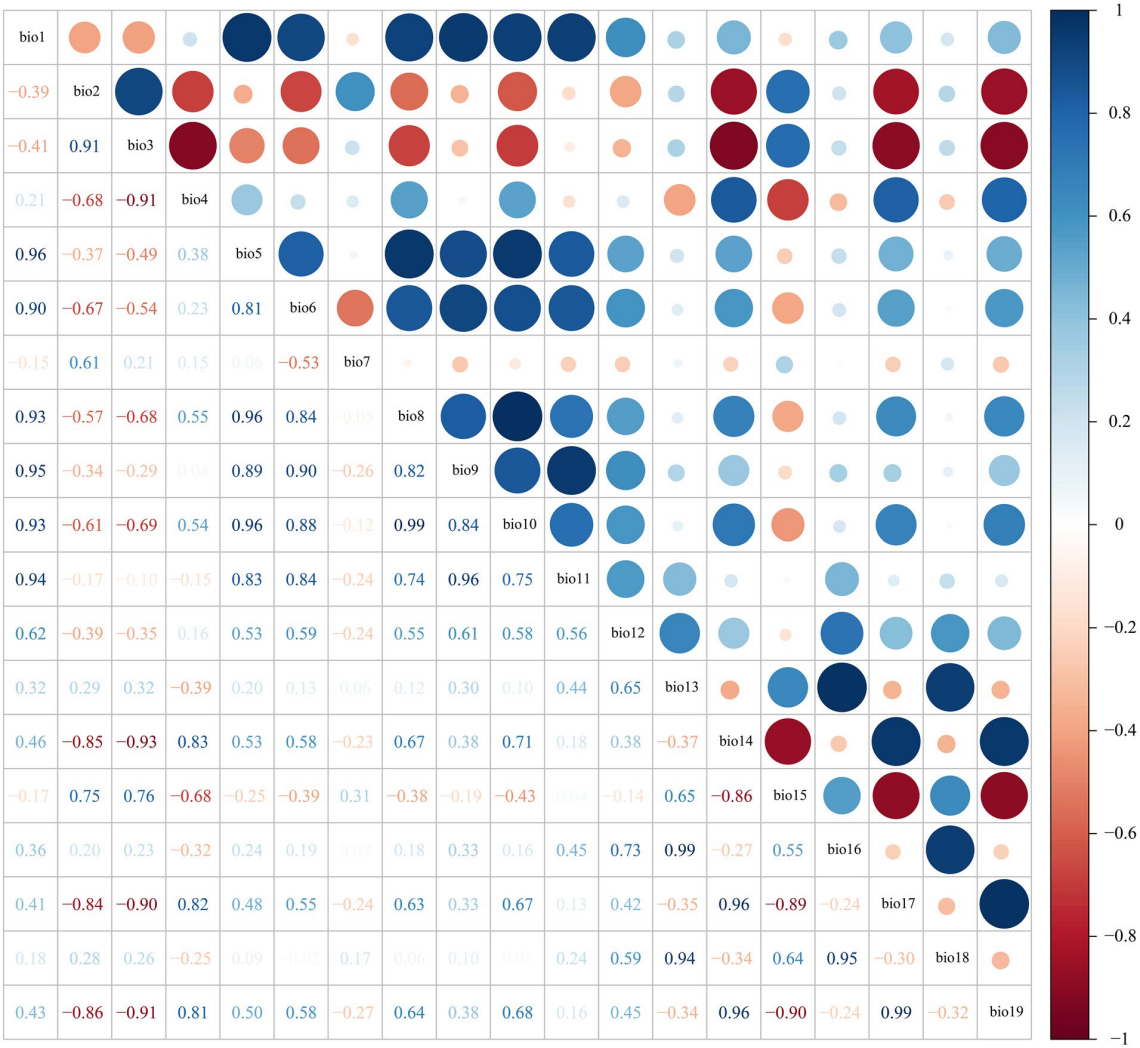
Correlation analysis of 19 environmental variables.

The spatial distribution of species on a large scale is primarily influenced by climatic conditions. Therefore, our research primarily examined the influence of climate on the distribution patterns of *T. chebula* . The bioclimatic data for the current, 2050s (2041–2060), and 2070s (2061–2080) utilized in this study are sourced from the global climatic database Worldclim (http://www.worldclim.org). The data have a pixel size of 2.5 arc‐minutes (−5 km). The provincial national vector map is obtained from Ministry of Natural Resources of the People's Republic of China (http://www.mnr.gov.cn/). In order to prevent overfitting resulting from the strong association between environmental factors, we examined the multicollinearity of 18 bioclimatic variables (Table S1) using Pearson correlation in IBM SPSS version 21 statistical software (Figure 3). If the coefficients of two variables exceed 0.80, the variable with lower ecological importance would be eliminated to enhance the accuracy of the model simulation. A total of eight environmental factors were chosen to create the potential distribution model for *T. chebula* (Table 1). The environmental variables were converted to ASCII format by ArcGIS 10.8 before modeling.

**TABLE 1:** The environment variables information.

Abbreviation	Environmental variables	Unit
bio1	Annual mean temperature	°C
bio2	Mean diurnal range	°C
bio4	Temperature seasonality	/
bio7	Temperature annual range	°C
bio11	Mean temperature of coldest quarter	°C
bio12	Annual precipitation	mm
bio15	Precipitation seasonality	/
bio18	Precipitation of warmest quarter	mm

### 2.3 Model Setting and Evaluation

The kuenm R package was utilized for thorough calibration, selection, final model development, and assessment of the MaxEnt model (Cobos et al. 2019). We used the “kuenm_occ_split” function to partition the occurrence data into subsets with a 75–25 ratio. Individual subsets were used for both model calibration and internal validation. A grand total of 1160 potential models were created by combining eight different groups of environmental predictors. The models included parameters that covered every possible combination of 40 different regularization multiplier values (ranging from 0.1 to 4 in increments of 0.1) and 29 combinations of feature classes for assessment (Hebbar et al. 2022; Li et al. 2023a). The candidate model's effectiveness was evaluated using various criteria including significance (assessed through partial ROC with 500 iterations and bootstrapping 50% of the data), omission rate (set at *E* = 5%), and model complexity (measured using AICc). Models with delta AICc values below 2 were chosen from the significant and low‐omission candidate models. The optimal parameter set was then used to construct the final model. This final model was subjected to 10 repetitions using the subsampling technique, where 75% of the data was randomly allocated to the training set and the remaining 25% to the test set. During each run, a random seed was applied to ensure different random partitions of the test and training sets, and a different random subset of the background was utilized. The jackknife approach was utilized to evaluate the significance of each environmental variable, construct response curves, and analyze how the diffusion probability of *T. chebula* responded to different environmental factors. Eventually, we adopted the average result of 10 repeated runs.

The MaxEnt model used the receiver operating characteristic (ROC) curve to assess the model's precision. The area under the curve, which signifies the area under the ROC curve, is viewed as a crucial measure of model accuracy. It is a plot of sensitivity versus specificity and is currently recognized as the most effective metric for assessing model performance. The AUC value ranges from 0.5 to 1.0, with a higher AUC indicating a more accurate prediction by the model. Typically, AUC scores are categorized into five levels: failing (0.50–0.60), poor (0.60–0.70), average (0.70–0.80), good (0.80–0.90), and excellent (0.90–1.00) (Hanley and McNeil 1982). In addition, commonly used model evaluation metrics include Kappa (Cohen 1960) and the True Skill Statistic (TSS) (Allouche, Tsoar, and Kadmon 2006) values. Therefore, we calculated the Kappa and TSS values to assess model performance, with both metrics ranging from −1 to 1. In practical applications, higher values of the AUC, Kappa, and TSS indicate greater accuracy and consistency in predictions.

### 2.4 Division of Suitable Areas

The average value outputted by the model was imported into ArcGIS, where a conversion tool was used to transform it from ASC format to Raster data. Employing natural breakpoints (Jenks), we segmented the forecasted outcomes. Based on Jenks' forecasted outcomes, the potential distribution predictions of *T. chebula* were classified into four groups: no suitability (0%–20%), low suitability (20%–40%), medium suitability (40%–60%), and high suitability (60%–100%).

## 3 Results

### 3.1 Model Optimization

**FIGURE 4:**
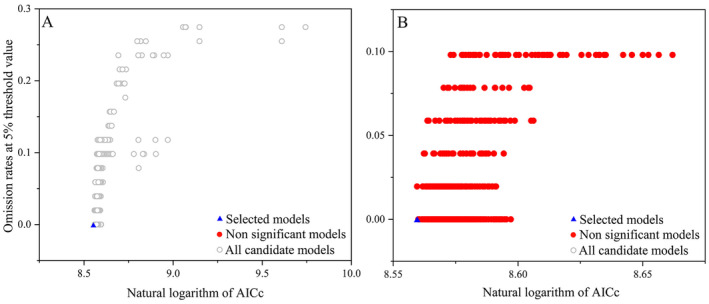
Omission rates and AICc values for all, non‐significant, and selected “best” candidate models for the tick (A) and the toad (B).

Optimizing the results according to the model, the 1160 resulting candidate models are statistically significant and outperform the null expectation (i.e., the model's predictions align better with the test occurrence data than predictions from randomly assigned points and regions) (Table 2). Among the candidate models, one specific model, M_1.5_F_qt, fulfills all the selection criteria for *T. chebula* (Figure 4). The selected model of M_1.5_F_qt has mean AUC ratio, omission rate at 5%, AICc, and delta AICc values of 1.89, 0.02, 5216.35, and 0, respectively (Table 2).

**TABLE 2:** Generated and selected candidate models and their fit and validation statistics.

Criteria	Number of models
All candidate models	1160
Statistically significant models	1160
Models meeting omission rate criteria	928
Models meeting AICc criteria	1
Statistically significant models meeting omission rate criteria	928
Statistically significant models meeting AICc criteria	1
Statistically significant models meeting omission rate and AICc criteria	1
Selected model	M_1.5_F_qt
Statistics of the selected model	
Mean AUC ratio	1.891
Omission rate at 5%	0.020
AICc	5216.35
Delta AICc	0

### 3.2 Model Performance and Contribution of Environmental Variables

**FIGURE 5:**
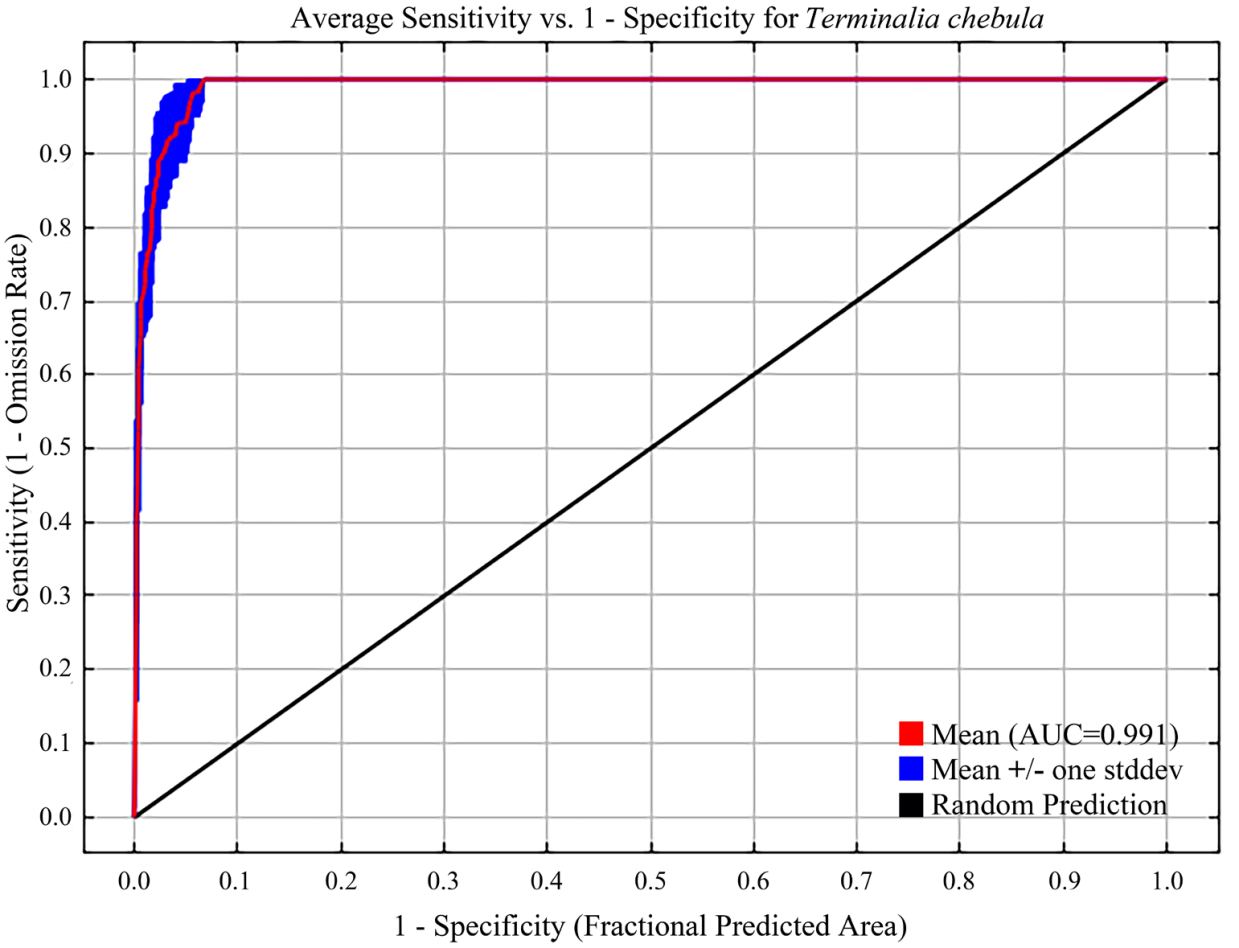
The receiver operating characteristic (ROC) curve. The values shown are the average of 10 replications. The red curve indicates training data, the blue curve indicates test data, and the black line indicates random prediction.

**FIGURE 6:**
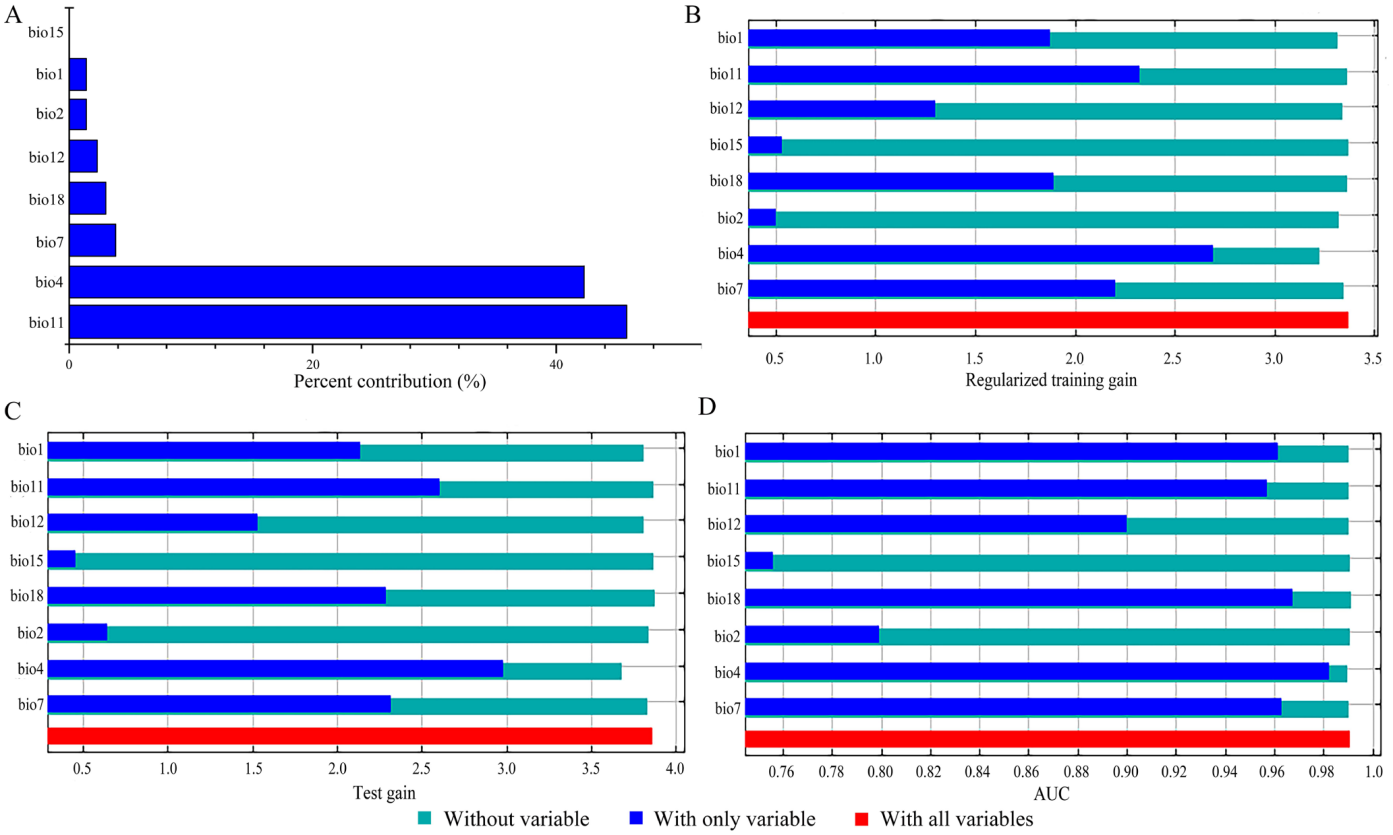
(A): Percent contributions of the environment variables in the MaxEnt models; (B), (C) and (D): The results of the jackknife test of variable importance.

As shown in Table 3, the AUC values were greater than 0.9, and the Kappa and TSS values exceeded 0.7. This indicates that the prediction model demonstrates high accuracy and consistency. The MaxEnt model's performance was assessed by measuring the area under the receiver operating characteristic (ROC) curve. The findings indicate that the accuracy of this approach in predicting the current *T. chebula* period is excellent (AUC mean = 0.991 ± 0.003, Figure 5). Variable contribution analysis and variable significance jackknife test results indicate that the bio11 and bio4 significantly influence the potential distribution of *T. chebula* (Figure 6). Bio11 (45.8%) has the greatest influence, followed by bio4 (42.3%) (Figure 6). The combined contribution rate of these two factors amounts to 88.1%. Bio7, bio18, bio12, bio2, bio1, and bio15 are other environmental factors that have influences of 3.8%, 3%, 2.3%, 1.4%, 1.4%, and 0, respectively (Figure 6). These six environmental variables do not have a significant impact on model prediction (< 4%). Temperature plays a vital role in predicting the potential distribution of *T. chebula* .

**TABLE 3:** The values of AUC, Kappa, and TSS.

Category	Value
AUC	0.991
Kappa	0.859
TSS	0.785

### 3.3 Response to Dominant Environmental Variables

**FIGURE 7:**
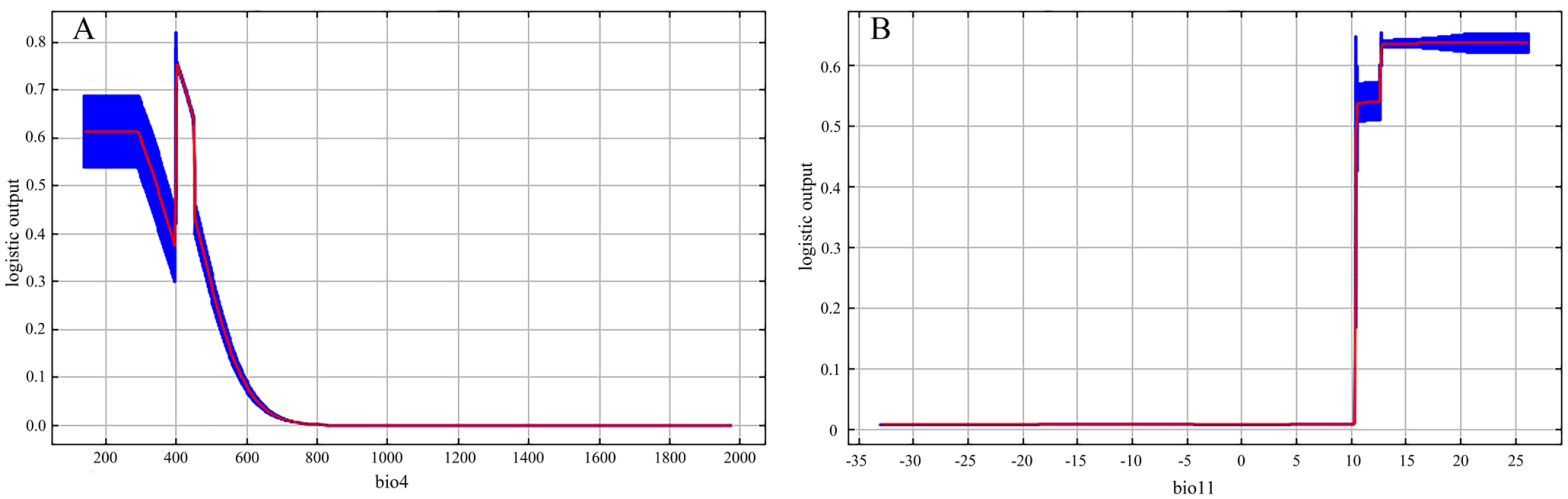
Response curves of climate suitability for the major environmental factors. (A) Temperature seasonality (bio4), (B) Mean temperature of coldest quarter (bio11).

These graphs depict the correlation between forecasted suitability and the chosen variable, showing how the anticipated probability of presence varies. With the increase in bio4, the probability of occurrence initially decreases, peaks at 400, and then decreases again (Figure 7A). Among the environmental factors, the bio11 remains at zero below 10°C, then increases with rising temperatures, reaches its peak at around 12.5°C, and then tends to stabilize with the increase of bio11 (Figure 7B). In other words, areas with temperature seasonality around 400 and higher average temperatures during the coldest month show higher suitability for *T. chebula* cultivation.

### 3.4 Current Distribution of *T. chebula*

**FIGURE 8:**
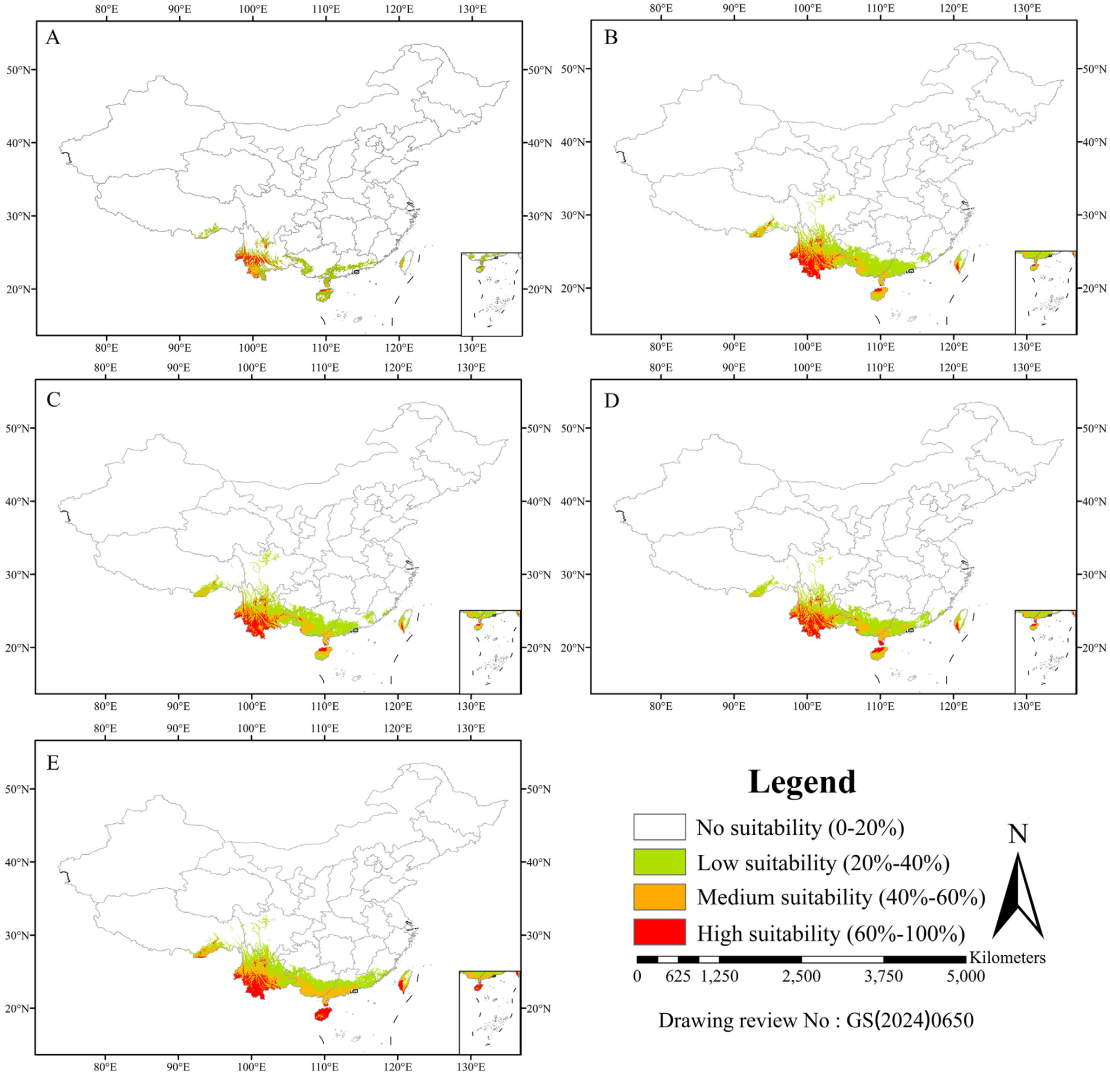
Suitable habitat distribution for *T. chebula* in China in the current and future under different climate change scenarios. (A) Current, (B) RCP4.5–2050s, (C) RCP4.5–2070s, (D) RCP8.5–2050s, (E) RCP8.5–2070s.

The model results illustrated in Figure 8A indicate that the species' distribution is predominantly focused in the southwestern regions of China. Under the current climate model, highly suitable habitat areas have been identified in Southwestern Yunnan and the northern part of Hainan. The areas evaluated as moderately and lowly suitable habitats mainly include the regions along the southern part of Yunnan, the western part of Guangxi, the eastern and southern parts of Guangdong, most areas of Hainan, and parts of Tibet and Sichuan in China. The total area of the currently suitable area is predicted to be 29.14 × 10^4^ km^2^, with 4.11 × 10^4^ km^2^ of high suitable habitat, 5.66 × 10^4^ km^2^ of medium suitability habitat, and 19.37 × 10^4^ km^2^ of low suitable habitat. In addition, the high suitability, medium suitability, low suitability, and no suitability areas account for 0.39%, 0.54%, 1.85%, and 97.21% of China's total land area, respectively (Table 4).

**TABLE 4:** Predicted suitable areas for *T. chebula* under current and future climatic conditions.

Decades scenarios	Suitability grade	Predicted area (10^4^ km^2^)	Proportion (%)
Current	No suitability	1015.86	97.21
	Low suitability	19.37	1.85
	Medium suitability	5.66	0.54
	High suitability	4.11	0.39
RCP4.5 2050s	No suitability	975.26	93.33
	Low suitability	39.03	3.74
	Medium suitability	20.36	1.95
	High suitability	10.35	0.99
RCP4.52070s	No suitability	971.79	92.99
	Low suitability	37.12	3.55
	Medium suitability	24.52	2.35
	High suitability	11.56	1.11
RCP8.5 2050s	No suitability	976.35	93.43
	Low suitability	37.99	3.64
	Medium suitability	19.52	1.87
	High suitability	11.15	1.07
RCP8.5 2070s	No suitability	966.23	92.46
	Low suitability	38.17	3.65
	Medium suitability	26.27	2.51
	High suitability	14.34	1.37

### 3.5 Future Distribution of *T. chebula*

To evaluate how the availability of suitable habitats for *T. chebula* may shift in response to present and future climate conditions (Figure 8), we analyzed the size of suitable habitat areas and the rate of change in these habitats (SHCR) for *T. chebula* across different projected scenarios. In comparison to the current distribution, there is a noticeable upward trend in the total area of suitable habitats. The suitable habitats will progressively spread from the southwest towards the east and north (Figure 8B–E). Climate predictions for the 2050s and 2070s are similar based on two Representative Concentration Pathways (RCPs): 4.5 and 8.5.

In the 2050s, under the RCP4.5, the total area of regions with high, medium, and low suitability for *T. chebula* is projected to increase by 0.6%, 1.41%, and 1.89%, respectively, compared to the current distribution (Table 4). The total area of suitable habitat in the 2050s under RCP4.5 is predicted to be 69.74 × 10^4^ km^2^, with 10.35 × 10^4^ km^2^ classified as high suitable habitat, 20.36 × 10^4^ km^2^ as moderately suitable habitat, and 39.03 × 10^4^ km^2^ as low suitable habitat area (Table 4). Under the RCP8.5, the total area of regions with high, medium, and low suitability for *T. chebula* is projected to increase by 0.68%, 1.33%, and 1.79%, respectively, compared to the current distribution (Table 4). The total area of suitable habitat in the 2050s under RCP8.5 is predicted to be 68.66 × 10^4^ km^2^, with 11.15 × 10^4^ km^2^ classified as high suitable habitat, 19.52 × 10^4^ km^2^ as moderately suitable habitat, and 37.99 × 10^4^ km^2^ as low suitable habitat area (Table 4).

In the 2070s, under the RCP4.5, the total area of high suitability, medium suitability, and low suitability for *T. chebula* under the RCP4.5 will increase by 0.72%, 1.81%, and 1.7%, respectively (Table 4). The total area of the suitable habitat in the 2070s under RCP4.5 is predicted to be 73.2 × 10^4^ km^2^, with 11.56 × 10^4^ km^2^ classified as highly suitable habitat, 24.52 × 10^4^ km^2^ as moderately suitable habitat, and 37.12 × 10^4^ km^2^ as low suitable habitat area (Table 4). Under the RCP8.5, the total area of high suitability, medium suitability, and low suitability for *T. chebula* under the RCP8.5 will increase by 0.98%, 1.97%, and 1.8%, respectively (Table 4). The total area of the suitable habitat in the 2070s under RCP4.5 is predicted to be 78.78 × 10^4^ km^2^, with 14.34 × 10^4^ km^2^ classified as highly suitable habitat, 26.27 × 10^4^ km^2^as moderately suitable habitat, and 38.17 × 10^4^ km^2^ as low suitable habitat area (Table 4).

## 4 Discussion

### 4.1 Reliability of Model Predictions

This research is the initial exploration of the effects of global climate change on the range and preferred environments of the plant species *T. chebula* in China, utilizing MaxEnt modeling. Studies related to this topic have verified that the size of the sample in species distribution data, as well as the concentration of distribution points, play a crucial role in influencing the precision of species model simulation outcomes (Chen et al. 2012; Wang et al. 2023b). Generally, as the sample size increases, the precision of species distribution model simulations improves. The rate of improvement diminishes over time until it stabilizes at the model's maximum accuracy (Chen et al. 2012). If the distribution points are overly clustered, the issue of overfitting due to the spatial autocorrelation of these points will be exacerbated, which will affect the simulation outcomes (Wang et al. 2023b; Yao, Han, and Lin 2023). In this research, 292 locations where *T. chebula* is found were collected. The relationship between these locations and environmental factors was examined and confirmed, resulting in a more focused dataset of 73 distribution points. This process helped reduce inaccuracies in modeling outcomes due to the limited sample size and significant multicollinearity among environmental variables.

The kuenm R package facilitates the optimization of the Maxent model by enabling adjustments to its parameters, thereby improving model performance (Cobos et al. 2019). By optimizing the Maxent model parameters, it can effectively simplify the model, enhance the alignment between predicted outcomes and observed reality, and precisely forecast species distribution (Cobos et al. 2019; Phillips and Dudík 2008). This model has been used to predict the distribution of *Zanthoxylum bungeanum* Maxim. (Zhuo et al. 2020), *Cocos nucifera* (L.) (Hebbar et al. 2022), *Pennisetum alopecuroides* (L.) (Xu et al. 2023), and *Davidia involucrata* Baill. (Wang et al. 2024). In our study, we used the kuenm R package to generate 1160 candidate models that included environmental predictors, regularization multipliers, and feature classes for the purpose of model calibration. The models were classified based on their importance, rate of omission, and complexity (Cobos et al. 2019). The best model selected for forecasting was M_1.5_F_qt. The MaxEnt model showed excellent accuracy in predicting the potential range of *T. chebula* under different climate scenarios, consistently achieving AUC values higher than 0.991. This level of performance indicates the model's effectiveness in forecasting the species' habitat suitability based on environmental variables. The findings are in agreement with earlier studies that focus on the distribution patterns of traditional Chinese medicinal herbs (Sharma et al. 2018; Shen, Yu, and Wang 2021; Wei et al. 2018; Yi et al. 2016), emphasizing the effectiveness of the MaxEnt approach in ecological modeling and species conservation efforts.

Although we utilized the ENMtools software to filter the distribution points of the initial data in order to minimize sampling bias, and employed the kuenm R package to optimize the Maxent model parameters and enhance prediction accuracy, small sample sizes in species distribution predictions may not adequately represent the true distribution of species. Consequently, MaxEnt may overfit the noise present in these limited datasets. The complexity of the model lies in its capacity to manage multiple environmental variables and their interactions. When faced with a small sample size, the model is susceptible to creating overly specialized distribution relationships (Phillips, Anderson, and Schapire 2006).

### 4.2 Dominant Environmental Variables

Our models predicting the potential distribution of *T. chebula* are only using data from its native regions, not from areas where it has been introduced. This indicates that the environmental conditions suitable for the species in the simulation are more in line with its actual habitat rather than its full potential range (Booth 2017). Previous studies have indicated that the actual habitat of a species is typically smaller than its full potential habitat (Soberón and Arroyo‐Peña 2017). The most critical factor determining population's regeneration and growth may be climate (Zhang, Li, and Du 2018). Climate plays a crucial role in shaping the distribution patterns of species. It regulates plant phenology and physiological processes, such as flowering and leaf deciduousness, as well as water usage, thereby influencing the spatial distribution of species (Manzoor, Griffiths, and Lukac 2021; Marchioro and Krechemer 2021; Pyšek et al. 2020). In particular, temperature and precipitation significantly affect plant growth, development, physiological metabolism, and the survival of species (Poncet et al. 2010). This exclusively study focuses on climate variables, including eight main variables: bio1, bio2, bio4, bio7, bio11, bio12, bio15, and bio18, and does not take into account other abiotic factors like soil composition, hydrogeology, altitude, and so forth. This research indicates that temperature‐related environmental factors have a more significant influence on the ideal habitat for *T. chebula* compared to precipitation‐related factors. The main environmental variables affecting the suitable habitat of *T. chebula* are bio11 and bio4 (Figure 6).

*Terminalia chebula* is a tropical and subtropical plant. Its flowering period occurs from May to June, while the fruits mature between November and December. The bio11 can impede the physiological activities of the fruits, leading to a reduction in the activities of enzymes such as sucrose synthase and amylase. This inhibition hinders sugar accumulation and adversely affects fruit quality. Additionally, low temperatures may suppress the expression of genes associated with anthocyanin synthesis and the activity of relevant enzymes, preventing the normal color change of the fruits. Furthermore, frostbite can occur, resulting in the softening and rotting of the fruits, as well as damaging to the seeds, which negatively impacts their germination ability and reduces seed vitality. Bio4 is a significant factor influencing plant phenological events and affecting the interactions between plants and other organisms. Large temperature fluctuations during the flowering and fruiting periods can compromise pollen viability, fertilization, and overall fruit development.

### 4.3 Potential Distribution and Migration

In the realm of global warming, the influence of environmental elements, particularly climate factors, on the distribution of species has emerged as a crucial area of study. Researchers studied the effects of climate change on species distributions (Moraitis, Valavanis, and Karakassis 2019; Wilson, Skinner, and Lotze 2019). The results indicate that *T. chebula* is mainly found in the southwestern Yunnan province, where it has the highest population density and a particularly suitable habitat (Figure 8A). Areas that are moderately suitable are usually located near highly suitable areas, while low suitability areas are more scattered. These findings align with the known distribution areas of *D. involucrate* (Wang et al. 2024) and *Glycyrrhiza inflata* Batal. (Du et al. 2024). The future spatial pattern and area conducive for the growth of *T. chebula* will undergo substantial changes compared to the current climate conditions. According to the future climate model, the habitat of *T. chebula* is expected to increase in size towards the northern and eastern regions. The distribution of the suitable habitat consistently shifts in response to climate change. As climate warming intensifies, the spatial location of the corresponding region will shift more significantly. The findings suggest that the suitable habitat for *T. chebula* is expected to shift towards more northern latitudes in the upcoming years, consistent with the outcomes reported in earlier studies (Guo et al. 2023; Wang et al. 2023b). It is evident that *T. chebula* , similar to subtropical plants such as *Magnolia wufengensis* (Linn.) Presl (Meng et al. 2021), *Camellia sinensis* (Zhao et al. 2021), and *Cinnamomum camphora* (L.) Presl (Li et al. 2023b), will migrate northward as temperatures rise in the future. This shift will increase the suitable habitat area in China, thereby enhancing the survival and reproduction of these plants.

### 4.4 Resources Conservation and Sustainable Use

The size of species distribution area is an important spatial feature closely related to species extinction, ecological invasion, and niche breadth. It is greatly significant for studying the origin, diffusion, and evolution of species. Amid the context of worldwide climate change, simulating and analyzing the predominant factors influencing the potential distribution area of species can offer a scientific foundation for the effective conservation and sustainable use of plant resources. The results show that climate change has a positive impact on the distribution of *T. chebula* . *T. chebula* , as a plant with medicinal value, primarily relies on wild collection to meet market demand, which can lead to significant damage to wild resources. In addition, the suitable habitat for *T. chebula* is expected to expand under future climate change; however, habitat suitability in certain areas may decline due to factors such as the destruction of wild resources, land use changes, and deforestation. Human activities are among the primary drivers of climate change. Ecosystem damage resulting from land use changes and deforestation may impede the natural migration of *T. chebula* , increase its survival pressures, exacerbate the impacts of extreme weather events, and further diminish the distribution of this species.

In order to protect and sustainably utilize the resources of *T. chebula* , we propose the following recommendations. One the one hand, it is crucial to preserve and protect wild *T. chebula* resources. Establishing protected areas in mountainous regions can minimize human intervention and contribute to the preservation plants. On the other hand, in order to prevent the decline of population genetic diversity, it is necessary to implement specific measures to minimize human‐induced damage and alterations to the natural habitat. Collecting wild germplasm resources of *T. chebula* for diversity assessment, particularly in regions at high risk of habitat loss, can aid in the preservation of core germplasm. Additionally, Governments should take more action to raise awareness about the protection of local medicinal plants and to prevent unsustainable land use and excessive logging. At the same time, conducting artificial breeding and cultivation outside the region is essential.

## 5 Conclusions

Evaluating how climate change impacts the potential range of *T. chebula* is crucial for the sustainable management and utilization of this tree species. Our study is the first to model the suitable habitats for *T. chebula* in China. This study demonstrates that the MaxEnt model, utilizing 73 occurrence records and eight environmental factors, can effectively simulate habitat distribution patterns of *T. chebula* . Furthermore, it is able to predict the main environmental factors affecting species distribution under current and future climate conditions. The research indicates that in the current climate model, the prime habitat for *T. chebula* is primarily found in southwestern Yunnan and the northern region of Hainan, covering a total area of 4.11 × 10^4^ km^2^. Precipitation of the bio11 and bio4 are the dominant environmental variables. The area of potentially suitable habitat increases northward and eastward under future climate scenarios.

## Author Contributions

**Zhang‐Hong Dong:** data curation (equal), formal analysis (equal), investigation (equal), methodology (equal), software (equal), validation (equal), visualization (equal), writing – original draft (equal), writing – review and editing (equal). **Hua Jiang:** data curation (equal), formal analysis (equal), investigation (equal), methodology (equal), software (equal), validation (equal), visualization (equal), writing – original draft (equal), writing – review and editing (equal). **Wei Zhang:** data curation (equal), investigation (equal), software (equal). **Jianhua Wu:** methodology (equal), validation (equal). **Yanping Yang:** data curation (equal), investigation (equal). **Taoming Yang:** software (equal). **Jiangping Zhao:** data curation (equal). **Cunzhen Luo:** investigation (equal). **Xiaoxia Yang:** data curation (equal). **Guilin Li:** investigation (equal), methodology (equal), supervision (equal), visualization (equal), writing – original draft (equal), writing – review and editing (equal).

## Conflicts of Interest

The authors declare no conflicts of interest.

## Supporting information


**Table S1.** Species distribution sample points.

**Table S2.** Modeling environment variables for potential geographic distribution of *T. chebula* .

## Data Availability

The spatial distribution data of *T. chebula* were from the Global Biodiversity Information Facility (GBIF; https://www.gbif.org/), Chinese Virtual Herbarium (CVH; https://www.cvh.ac.cn/), National Specimen Information Infrastructure (NSII; http://www.nsii.org.cn/). Climate data form Worldclim (http://www.worldclim.org).
